# Outcome of cryopreserved-thawed embryo transfer in the GnRH agonist versus antagonist protocol

**Published:** 2012-07

**Authors:** Maryam Eftekhar, Razieh Dehghani Firouzabadi, Hesamoddin Karimi, Elham Rahmani

**Affiliations:** 1 *Research and Clinical Center for Infertility, Shahid Sadoughi University of Medical Sciences, Yazd, Iran. *; 2 *Obstetrics and Gynecology ward, Bushehr University of Medical Sciences, Bushehr, Iran.*

**Keywords:** *GnRH agonist*, *GnRH antagonist*, *Cryopreserved*, *Embryo transfer*, *Pregnancy outcome*

## Abstract

**Background:** GnRH agonist and antagonist were developed to control the premature release of LH surge. There is some difference between two protocols.

**Objective: **We compared the outcome of frozen-thawed embryo transfer in infertile women who used GnRH agonist or antagonist protocol for previous COH cycle and evaluation of any adverse effect of GnRH antagonist on oocyte and embryo.

**Materials and Methods:** The study group included all infertile women who referred to Yazd Research and Clinical Center for Infertility. Overall 20-35 years old women who were candidate for frozen-thawed embryo transfer with regard to inclusion and exclusion criteria were participated in the study. The patients based on previous control ovarian stimulation (COH) protocol divided in to two groups: GnRH agonist long protocol (n=165) and GnRH antagonist multiple dose protocol (n=165). Frozen-thawed embryos were transferred after endometrial preparation in both groups. Main outcome measures were: implantation, chemical and clinical pregnancy rate.

**Results:** The implantation and clinical pregnancy rate following cryopreserved embryo transfer in GnRH agonist group and antagonist group were 16.3% vs. 15.7% (p=0.806) and 38.1% (63/165) vs. 36.9% (61/165) (p=0.915) and chemical pregnancy rate was 44.8% (74/165) vs. 43.6% (72/165) (p=0.915) respectively.

**Conclusion:** There was no statistically difference between two groups in terms of implantation and pregnancy rate. Although pregnancy rate in fresh embryo transfer in antagonist cycles was lower than agonist groups, Therefore decrease in these parameters might be due to detrimental effect of GnRH antagonist on the endometrium, not embryo or oocyte.

## Introduction

GnRH agonist has been the standard protocol for ovulation induction in in vitro fertilization cycles for 20 years ago. However, GnRH agonist has some complication including: estrogen deprivation symptom, need more gonadotropin consumption, ovarian cyst formation. Pituitary function was not immediately returned following GnRH agonist discontinuation. 

After the discovery of GnRH (Gonadotropin Releasing Hormone) agonists and their use in pituitary desensitization, cycle cancellation due to premature LH surge was significantly decreased. During recent decades a new generation of GnRH antagonist have been introduced which can competitively block GnRH receptors and cause rapid LH surge inhibition (1-4). In some study GnRH agonist have been compared with GnRH antagonist for COS in infertile patients as the decrease of pregnancy rate in cycles using GnRH antagonist protocol was reported (5, 6). But decreasing of implantation and pregnancy rates following use of GNR antagonists are still controversial (7, 8). Lower pregnancy rate following antagonist protocol may be due to GnRH receptors which have been discovered in extra pituitary tissues including ovary, endometrium, myometrium and embryo. 

These receptors may inhibit by the extra pituitary GnRH antagonist effects and leading to decrease pregnancy rate. Decreasing of pregnancy rate due to detrimental effect of GnRH antagonist on oocyte quality or endometrial receptivity is still debate (7, 9-13). Transfer of frozen-thawed embryo makes a possibility to eliminate any adverse effect of GnRH antagonist on endometrium that may cause lower pregnancy rate. Therefore assessment of ART outcome using cryopreserved-thawed embryos, provide an opportunity to compare the effect of GnRH antagonist and agonist protocols in the same situation. 

In this study, we evaluate ART outcomes in frozen-thawed embryo transfer cycles in the two groups of patients who have used GnRH agonist or antagonist in previous controlled ovarian hyper stimulation (COH).

## Materials and methods

This retrospective cohort study was conducted at Yazd Research and Clinical Center for Infertility, Shahid Sadoughi University of Medical Sciences, between January 2009 and June 2011. The study was approved by ethics committee. 330 couples were participated in this study. All women had previously undergone controlled ovarian stimulation with standard long agonist protocol or antagonist protocol and in-vitro fertilization (IVF) or intracytoplasmic sperm injection (ICSI) with embryo cryopreservation was done for them. All of the patients failed fresh embryo transfer, and then were candidate for cryopreserved embryo transfer. 

In group I (agonist group), Decapeptyl (Decapeptyl® 0.1 mg, Ferring , Germany) was started 0.1 mg per day subcutaneously from previous mid-luteal phase. Decapeptyl dose was decreased to 0.05 mg/day on the first day of menstrual bleeding and continued until the day of HCG injection. Ovarian stimulation was done from day 2 of menstrual cycle with daily administration (150 IU) of human recombinant follicle-stimulating hormone (Gonal-f, serono, Aubnne, Switzerland) and continued until the day of HCG injection. Ovarian response was monitored using serial ultrasound examination.

Ovarian stimulation was started in group II (antagonist group) from second day of menstrual cycle with (150 IU) of human recombinant follicle-stimulating hormone (Gonal-f, serono, Aubnne, Switzerland) monitoring by serial vaginal sonography was done. When dominant follicles reached to 14 mm in mean diameter, 0.25mg/day of GnRH antagonist (cetrotide, sereno) was started and continued until the day of HCG injection. In both groups when at least two follicles with a mean diameter of 17 mm or one leading follicle was larger than 18 mm, were observed 10000 IU HCG (Pregnyl, Organon, Netherland) was injected. For all of the patients’ endometrial thickness and serum E_2_ levels were measured in the day of HCG injection. 

A three-layered endometrium was seen in all of the patients. Oocyte retrieval was done 34-36 hours after HCG injection, using a 17-gauge needle under vaginal ultrasonography guidance and conventional IVF or intracytoplasmic sperm injection (ICSI) was done appropriately.

More than 3 embryos were not transferred in fresh cycles and all the excess embryos with <30% fragmentation was cryopreserved by vitrification method. Women with age >35 years, BMI >30, previous ovarian hyper stimulation syndrome, history of D.M. and thyroid disease and history of sever endometriosis were excluded from the study. Oocyte donation cycles were excluded from the study. Only patients that had implantation failure after fresh embryo transfer were participated in the study. Frozen embryo cycle was used at least 2 months after fresh cycle. 

Endometrial preparation in both group was similar, estradiol valerate (Estradiol valerate, Aburaihan CO, Tehran, Iran) was taken orally at the dose of 6 mg per day from the second day of menstrual cycle. In day 13 of cycle, an ultrasound examination was performed. It was used to assess endometrial thickness. When the endometrial thickness reached more than 8 mm in diameter, 100 mg progesterone in oil (progesterone, aburaihan, CO, Tehran, Iran) was injected daily. Estradiol and progesterone consumption were continued until the documentation of fetal heart activity by ultrasound. 

Thawing of the embryos in both groups was performed 2 days after beginning of progesterone injection. In both group embryos transfer 1 day after thawing by using a labotect catheter (Labotect, Gottingen, Germany). Embryo quality was assessing using the modified cumulative embryo score (14). Good quality embryo was transferred by one of the expert specialist. Primary outcome was defined as ongoing pregnancy. Implantation was defined by number of gestational sacs per number of transferred embryos. Chemical pregnancy was defined by serum β-hCG >50 IU/L, 12 days after embryo transfer, clinical pregnancy was defined by observation of fetal heart activity by transvaginal ultrasonography 5 weeks after positive β-hCG. 

**Statistical analysis**

The student t-test was used to continuous variable and chi-square test was used to compare attributive variables. We used statistical software SPSS Version 16 (SPSS Inc., Chicago, USA). Our statistical significant was set at p<0.05. 

## Results

Three hundred thirty couples were participated in this study and patients were divided into two groups 165 patients in agonist group and 165 patients in antagonist group. Demographic and infertility characteristic are shown in table I, female age, duration of infertility, basal FSH, BMI and etiology of infertility were similar in both groups. The cycle characteristics and outcome of ART are showed in table II. No statistically differences were reported in implantation, chemical and clinical pregnancy.

**Table I:** Basic characteristics of patients of patients in two groups

**Variables**		**Agonist group** ** (n=165)**	**Antagonist group** **Mean±SD (n=165)**	**p-value**
Female age (years)*		29.8 ± 4.48	29.5 ± 4.55	0.942
Duration of infertility (years)*		8.65 ± 4.67	8.12 ± 3.80	0.266
Basal FSH (IU/L)*		5.61 ± 2.0	5.68 ± 1.72	0.812
BMI (Kg/m^2^)*		23.45 ± 3.4	23.65 ± 2.3	0.336
Etiology of infertility, n (%)				0.099
	Male	74 (44.8%)	60 (36.4%)	
	PCO	55 (33.3%)	54 (32.7%)	
	Tubal	9 (5.5%)	9 (5.5%)	
	Unexplained	18 (10.9%)	21 (12.7%)	
	Mix	9 (5.5%)	21 (12.7%)	

* (Mean±SD)

**Table II:** Cycle characteristic and ART outcome in two groups

**Variables**	**Agonist group** **(n=165)**	**Antagonist group** **(n=165)**	**p-value**
Endometrial thickness (mm)*	9.45 ± 1.1	9.51 ± 1.66	0.639
Duration of estradiol consumption (day)*	17.15 ± 1.37	17.32 ± 1.10	0.219
No. of embryos transferred*	2.65 ± 0.73	2.92 ± 0.9	0.32
Implantation rate (%)	16.3%	15.7%	0.806
Chemical pregnancy rate, n (%)	74 (44.8%)	72 (43.6%)	0.825
Clinical pregnancy rate, n (%)	63 (38.1%)	61 (36.9%)	0.915

* (Mean±SD)

## Discussion

GnRH agonist are used as the standard treatment protocol in controlled ovarian stimulation cycles, but in recent years by introducing GnRH antagonists, new horizons was created in the treatment of infertile patients. There are several advantages over GnRH antagonists: rapid suppression of the pituitary due to competitive inhibition of GnRH receptor, their effect is rapid and dose-

dependent, have no initial flair effect, decreased length of treatment cycle, reduce the amount of gonadotropin consumption, reduce OHSS risk, reduce estrogen deprivation symptoms (1-4, 8, 15, 16). 

In this study, we detected no difference in cryopreserved-thawed implantation, chemical and clinical pregnancy rate, between GnRH agonist and antagonist protocol. Previous studies on cryopreserved-thawed obtained similar findings, in a retrospective study on 406 infertile women found no significant difference in the pregnancy rate per thawed cycles and cumulative live birth rate but in term of post thawed blastocyst survival, GnRH agonist group was higher (17). Bahçeci *et al* published 714 infertile patients who transferred fresh embryo or frozen-thawed embryo showed no difference between agonist or antagonist groups in implantation and pregnancy rate in cryopreserved-thawed group but in fresh embryo transfer group, implantation and pregnancy rate was significantly different (8).

Some studies represented that GnRH antagonist can decrease ovarian paracrine activity by decreasing in insulin-like growth factor (IGF) and epidermal growth factor (EGF) biosynthesis, that are essential for folliculogenessis (7, 18-20), but another authors proposed that GnRH antagonists do not have detrimental effect on ovarian steroidogenesis or IGF biosynthesis and also represented that the intrafollicular levels of IGF-I and EGF do not seem to be influenced by the GnRH antagonist (21, 22).

In recent studies the effect of antagonists on the endometrium has been investigated expression of several growth factors and their receptors on the endometrium that seems to be effective in implantation (i.e., transforming growth factor, fibronectin and L-selectin) were investigated and demonstrated that GnRH analogues alters the expression of transforming growth factor-β (TGF-β) and receptors in endometrial cells and also GnRH analogues and TGF-β through MAPK/ERK Lead to changes in fibronectin expression in endometrial cells, a molecular mechanism that could influence embryo implantation (23).

Matrix metalloproteinase (MMP) and their specific inhibitors, role in Trophoblastic cell invasion into the endometrium and therefore they are important to implantation. GnRH Increases expression of MMP-9 and MMP-2, but GnRH antagonist inhibits these enzymes and therefore can disrupt in implantation (24).

GnRH antagonists as agonist are effective in inhibiting LH surge. Since the GnRH receptors were discovered in tissues outside the pituitary including: ovary, endometrium, myometrium and embryo, concerns have been increased about the detrimental effects of GnRH antagonists on extra pituitary tissue (9-12, 25). In several study these extra pituitary effect proposed as the cause of lower pregnancy rate in GnRH antagonist protocol but it is not obvious that witch extra pituitary effect of GnRH antagonist could be the main reason for a lower pregnancy rate. These concerns are according to several in-vitro studies suggesting decreased biosynthesis of growth factors caused by local action of GnRH antagonists (7, 26).

GnRH Antagonist effect on the expression of HOXA10 genes in endometrium which is an important regulator of endometrial receptivity In comparison with GnRH agonist it was demonstrated that in GnRH antagonist group HOXA10 expression reduced in endometrial stromal cells (27).

Frozen-thawed embryo transfer making possible a model to eliminate any detrimental effect of GnRH antagonist on endometrium that may cause lower pregnancy rate. Therefore assessment the cryopreserved-thawed outcomes provide an opportunity to evaluate the effect of GnRH antagonist on oocyte and embryo. These findings suggest that the lower ART outcome in GnRH antagonist protocol seems to be due to detrimental effect of GnRH antagonist on endometrium not embryo or oocyte. According to result of our study, we also completely agree with previous study witch demonstrated that GnRH antagonist have no adverse effect on oocyte or embryo (3, 4, 8, 13, 28-30).

In these studies the effect of antagonists on the results of ART cycles and their likely effects on the endometrium or embryo have been studied. Considering the results of these studies less success in ART cycles using GnRH antagonists compared with agonists does not seems to be due to adverse effects on the oocytes or embryo. The Main limitation of our study is its retrospective, despite this; we have shown that the two groups were similar in base line characteristics parameters: female age, BMI, basal FSH, cause of infertility, type of infertility, duration of infertility and number of embryo transfer.

## Conclusion

In conclusion, pregnancy rate in fresh embryo transfer in antagonist cycles was lower than agonist groups. Therefore, decline in these parameters might be due to unfavorable effect of GnRH antagonist on the endometrium, not embryo or oocyte. GnRH antagonists are effective as agonists in outcome of cryopreserved-thawed embryo transfer in terms of implantation and pregnancy rates.

## Footnote

***This article was extracted from M.D. thesis.***
